# Sources of Calcium at Connexin 36 Gap Junctions in the Retina

**DOI:** 10.1523/ENEURO.0493-22.2023

**Published:** 2023-08-16

**Authors:** Yuan-Hao Lee, W. Wade Kothmann, Ya-Ping Lin, Alice Z. Chuang, Jeffrey S. Diamond, John O’Brien

**Affiliations:** 1 Richard S. Ruiz, Department of Ophthalmology and Visual Science, McGovern Medical School, University of Texas Health Science Center at Houston, Houston, Texas 77030; 2 Synaptic Physiology Section, National Institute of Neurological Diseases and Stroke, Bethesda, Maryland 20892; 3 MD Anderson Cancer Center UTHealth Graduate School of Biomedical Sciences, Houston, Texas 77030

**Keywords:** calcium, Connexin 36, electrical synapse, retina, SRRF

## Abstract

Synaptic plasticity is a fundamental feature of the CNS that controls the magnitude of signal transmission between communicating cells. Many electrical synapses exhibit substantial plasticity that modulates the degree of coupling within groups of neurons, alters the fidelity of signal transmission, or even reconfigures functional circuits. In several known examples, such plasticity depends on calcium and is associated with neuronal activity. Calcium-driven signaling is known to promote potentiation of electrical synapses in fish Mauthner cells, mammalian retinal AII amacrine cells, and inferior olive neurons, and to promote depression in thalamic reticular neurons. To measure local calcium dynamics *in situ*, we developed a transgenic mouse expressing a GCaMP calcium biosensor fused to Connexin 36 (Cx36) at electrical synapses. We examined the sources of calcium for activity-dependent plasticity in retina slices using confocal or Super-Resolution Radial Fluctuations imaging. More than half of Cx36-GCaMP gap junctions responded to puffs of glutamate with transient increases in fluorescence. The responses were strongly dependent on NMDA receptors, in keeping with known activity-dependent signaling in some amacrine cells. We also found that some responses depended on the activity of voltage-gated calcium channels, representing a previously unrecognized source of calcium to control retinal electrical synaptic plasticity. The high prevalence of calcium signals at electrical synapses in response to glutamate application indicates that a large fraction of electrical synapses has the potential to be regulated by neuronal activity. This provides a means to tune circuit connectivity dynamically based on local activity.

## Significance Statement

Electrical synapse plasticity is controlled at the level of the individual synapse, and several mechanisms of plasticity depend on the presence of calcium. Tools available to researchers to study calcium dynamics generally provide a spatially diffuse view, without single-synapse resolution. We have developed a transgenic mouse that reports calcium dynamics at individual Cx36 electrical synapses, enabling investigators to study calcium microdomain dynamics in circuits of interest. Preliminary studies in retina reveal that calcium microdomains are dynamic at a large fraction of electrical synapses, demonstrating extensive potential for activity-dependent plasticity. Such plasticity is likely to be the norm for electrical synapses rather than an exception limited to a few studied circuits.

## Introduction

All mature neurons operate in networks functionally connected to other neurons by synapses. Chemical and electrical synapses generally have different functions within these networks. Although there are numerous important exceptions, chemical synapses generally form inhibitory or excitatory connections between neurons of different type, whereas electrical synapses form connections between cells of the same type. In this way, electrical synapses play important roles in establishing networks of neurons.

Electrical synaptic plasticity is a central element of many adaptive processes that optimize circuit function under changing conditions (Haas et al., 2016). Sensory systems must encode information over an enormous range of signal intensities. In the retina, the intensity of incident light may range over 10 orders of magnitude during the day, but encoding of retinal output in spike rates of retinal ganglion cells spans only about two orders of magnitude. Adaptation at many levels contributes to the signal compression that makes this coding possible. Electrical synapse plasticity contributes to both large-scale switching from high-sensitivity rod pathways to lower-sensitivity cone pathways (Ribelayga and O'Brien, 2017), as well as to more temporary adaptation to contrast (Shi et al., 2020). Motor control systems also depend on dynamic tuning of electrically coupled circuits in the inferior olive nucleus and the cerebellum to correct errors in movement timing (Schweighofer et al., 2013). In a similar manner, the thalamic reticular nucleus is thought to focus attention on certain streams of sensory information through dynamic adjustment of electrically coupled networks (Haas and Landisman, 2011; Coulon and Landisman, 2017). Such changes in coupling could be a general mechanism to regulate high-order processes in the brain (Pernelle et al., 2018).

In several known examples, electrical synapse plasticity depends on calcium and is associated with neuronal activity. Calcium-driven signaling promotes potentiation of electrical synapses in fish Mauthner cells (Pereda et al., 1998; Smith and Pereda, 2003), a motor neuron that controls the escape swimming reflex. Similar potentiation by a calcium signal has been observed in mammalian retinal AII amacrine cells (Kothmann et al., 2012) and inferior olive neurons (Turecek et al., 2014). In contrast, calcium signaling promotes depression in thalamic reticular neurons (Haas and Landisman, 2011; Sevetson et al., 2017). Calcium signaling has also been found to promote activity-dependent potentiation of innexin-based electrical synapses in a motor circuit in the leech (Welzel and Schuster, 2018), revealing that this type of signaling is both ancient and conserved through a broad phylogenetic range of the animal kingdom. These observations reveal that sensory, motor, and cognitive functions can all be modified by changes in electrical synapses instigated by calcium signaling.

Because of the widespread influence of calcium signaling on electrical synapse function, it would be useful to have the ability to study these calcium signals in real time at electrical synapses. To make this possible, we developed a transgenic mouse expressing a GCaMP calcium biosensor fused to Connexin 36 (Cx36) at electrical synapses, which enables the measurement of local calcium dynamics *in situ*. This study presents validation of this model as well as a survey of the sources of calcium for activity-dependent plasticity in retina slices. We find that a large fraction of Cx36-GCaMP gap junctions experience transient increases in local calcium concentration when stimulated with glutamate and that the responses depend both on NMDA receptors and voltage-gated calcium channels. This reveals that a dynamic calcium microenvironment at electrical synapses is widespread in the retina, providing a substrate for activity-dependent plasticity.

## Materials and Methods

### Development of the Cx36-GCaMP transgene

We customized the pBluescript-based vector pI-SceI (a gift from Jochen Wittbrodt; Thermes et al., 2002) by cutting the vector with SacII and NotI and inserting a linker comprising the 5′ phosphorylated oligonucleotides ACCTCGAGGACTTAAGA and GGCCTCTTAAGTCCTCGAGGTGC. This added restriction sites AbsI and AflII to assist with our specific cloning requirements while removing the parent restriction sites. The new vector was termed pBS-ISAA. Cx36-GCaMP (plasmid #123604, Addgene; Moore et al., 2020) was cut with BamHI and AflII to excise a fragment containing the GCaMP3-tagged Cx36 exon 2 and the SV40 polyadenylation sequence of the plasmid; this fragment was cloned into pBS-ISAA. A 5.3 kb fragment of the *Cx36* (*Gjd2*) gene containing intron 1, exon 1, and 4 kb of upstream sequence was amplified from mouse genomic bac RP23-230H3 (BACPAC) with primers CTTGATATCGAATTCTCCAGTCAGGGAACGTGTAGC and ACCACAGTCAACAGGATCCTGAACAGAGGAAGAGG using Phusion DNA polymerase (New England Biolabs). The amplified fragment was cloned into the Cx36 exon2-GCaMP clone in pBS-ISAA cut with BamHI and EcoRI using Cold Fusion Cloning Kit (System Biosciences), recreating the natural exon 1-intron 1-exon 2 structure of *Cx36*.

To test for proper splicing and gap junction formation of the construct, we cut the full-length Cx36 promoter-Cx36-GCaMP clone with EcoRV and SmaI, removing most of the promoter fragment, and resealed the plasmid. We then cut the entire insert out with AbsI and transferred it to pCAGEN (plasmid #11160, Addgene; Matsuda and Cepko, 2004). Transfection of this construct into HeLa cells (catalog# CCL-2, ATCC; RRID:CVCL_0030) resulted in expression of GCaMP-tagged Cx36 that formed gap junctions between cells, verifying that the construct was spliced and trafficked properly (data not shown).

### Development of Cx36-GCaMP transgenic mice

All experimental procedures described in this study comply with the U.S. Public Health Service policy on humane care and use of laboratory animals and the National Institutes of Health *Guide for the Care and Use of Laboratory Animals* and were approved by the Institutional Animal Care and Use Committees under protocols HSC-AWC-16–0063 at the University of Texas Health Science Center at Houston and ASP 1220 at the National Institute of Neurologic Disorders and Stroke. Mice were used in this study and maintained on a daily 12 h light/dark cycle. The full-length Cx36 promoter-Cx36-GCaMP clone was linearized with I-SceI and sent to the transgenic mouse core facility at the University of Texas Health Science Center at Houston for blastocyst injections in C57BL/6 background, embryo implantation, and rearing filial (F)0 pups. The F0 founder animals were crossed with C57BL/6J mice (Jackson Laboratory; RRID:IMSR_JAX:000664) and offspring screened for the transgene by PCR of tail DNA with primers CAGGACTCAGGACAGTGACTCTGCCTATG and GCGGCGGTCACGAACTCC. Several lines of mice were propagated from founders that displayed germline transmission of the transgene. Both female and male mice were used for the experiments described in this report.

### Immunofluorescence staining and confocal microscopy

Cryostat sections of retina, brain, and pancreas from Cx36-GCaMP mice were used for the assessment of protein expression levels and localization. Mice were anesthetized with isoflurane and killed by cervical dislocation. For frozen sections of retina or pancreas, freshly dissected tissues were fixed with 4% formaldehyde (Electron Microscopy Sciences) in 0.1 m PB, pH 7.5, for 30 min and then washed with PBS. Fixed tissues were cryoprotected overnight with 30% sucrose in 0.1 m PB, embedded in optimum cutting temperature compound (Sakura Finetek), and sectioned vertically with a cryostat. For frozen sections of brain, anesthetized mice were arterially perfused with PBS, followed by 2% formaldehyde in PBS for 20 min. Whole brains were then dissected out and further immersion fixed in 2% formaldehyde in 0.1 m PB for 5 h. Fixed brains were cryoprotected, embedded, and sectioned as for retina and pancreas. The cryostat sections of retina and brain were blocked in 10% donkey serum (Jackson ImmunoResearch) in PBS and 0.3% Triton X-100 (PBST) for 1 h and incubated with primary antibodies diluted in 5% donkey serum in PBST overnight at room temperature (RT). For cryostat sections of pancreas, an additional antigen retrieval step was included. Sections were incubated in 10 mm sodium citrate, pH 6.0, at 100°C for 1 h, cooled to RT in the same buffer for an additional 1 h, and washed with PBS. Slides were then blocked and probed as for other tissues. Antibodies used include mouse anti-Cx35/36 (catalog #MAB3045 Millipore-Sigma; RRID:AB_94632), goat anti-Cx36 (catalog #sc-14904, Santa Cruz Biotechnology; RRID:AB_2111311), rabbit anti-insulin (catalog #sc-9168, Santa Cruz Biotechnology; RRID:AB_2126540), and Alexa Fluor 488–conjugated rabbit anti-GFP (catalog #A-21311, Thermo Fisher Scientific; RRID:AB_221477). Secondary antibodies were made in donkeys and sourced from Jackson ImmunoResearch. Sections were coverslipped with Vectashield mounting medium with DAPI (Vector Laboratories) before imaging. Samples were imaged on a Zeiss LSM 780 confocal microscope with 63×/1.4 NA or 40×/1.4 NA oil objectives. For localizing and quantifying the expression levels of intrinsic GCaMP, or immunostains of Cx36 or GFP, three consecutive confocal slices with a total thickness of 0.6 μm in the middle of the inner plexiform layer (IPL) were selectively imaged and flattened with a maximum intensity projection using Zeiss Zen 2.3 software.

### Immunofluorescence image processing and data quantification

Images for display in figures were enhanced for contrast and brightness with Adobe Photoshop (RRID:SCR_014199) or Fiji software (RRID:SCR_002285; Schindelin et al., 2012). Image quantification was performed using Fiji. Regions of interest (ROIs) selection was made through thresholding on the fluorescence channels of Cx36 and anti-GFP immunolabeling with a standard 15% threshold. The selections of detected binary objects were further modified by the editor function Wellspring to isolate individual gap junctions and limited to objects larger than 1 pixel and smaller than 50 pixels. The coexpression of Cx36 and GCaMP was assessed by measuring the fluorescence intensity of the nonselected channel in each ROI. Positive colocalization was scored if the measured intensity exceeded a threshold greater than the summed level of the background average plus two times the background SD in that channel.

### Solutions

Eight puffing and three bathing solutions were used for detecting the transient puffing responses during live imaging. All puffing and bathing solutions were made in Ames medium (Thermo Fisher Scientific) and complemented with none, 20 μm (*R*)-CPP (3-((*R*)−2-carboxypiperazin-4-yl)-propyl-1-phosphonic acid; Tocris Bioscience), 100 μm picrotoxin (Tocris Bioscience), 20 μm Nifedipine (Tocris Bioscience), or 100 μm CdCl_2_. Puffing solutions were completed by adding either glutamic acid at 1 mm or NMDA at 10 μm along with 1 mm glycine (Sigma-Aldrich) or AMPA at 100 μm. In addition, all bathing and puffing solutions were supplemented with 10 μm l-AP4 (l -(+)−2-amino-4-phosphonobutyric acid; Tocris Bioscience) to suppress light-driven On pathway responses.

### Retina dissection and slicing for transient puffing responses

Mice were anesthetized with isoflurane and killed by cervical dislocation. After eyeball enucleation, the retina was dissected in oxygenated Ames medium and mounted photoreceptor side up on black nitrocellulose filter paper (EMD Millipore). The retina was sliced with a homemade micrometer-controlled positioning stage and slicing blades (Feather Double Edge) into 150 μm slices. The retina and slices were maintained submerged in oxygenated Ames medium throughout the procedure. Retina slices were mounted in perfusion chambers (Warner Instruments) and perfused continuously with oxygenated Ames medium using a peristaltic pump (Instech Laboratories) at 2 ml/min.

Borosilicate glass puffing pipettes were fabricated with a tip width of 20 μm and a taper of 3 mm with a Flaming–Brown pipette puller (Sutter Instrument). Pipettes were loaded with puffing solutions and localized close to retinal IPL within the field of view. Puff solutions were administered 10 s after the start of image acquisition at the injection pressure of 0.2 psi for 2 s using a Pico-Injector (Harvard Apparatus).

### Live imaging and image processing

Retina slices were imaged live with visible light excitation and l-AP4 in the bath to suppress excitatory On pathway responses. Preliminary experiments were performed with a Zeiss LSM510 confocal microscope using a 40×/1.0 NA water immersion objective. Subsequent experiments were performed in an upright Olympus BX51-WI conventional light microscope. Slices were viewed with 4× dry or 40×/0.8 NA water immersion objectives. GCaMP fluorescence was excited with a Xenon lamp (Sutter Instrument) with a 470BP40 filter and detected through a 525BP50 filter. Images were captured with an ANDOR iXon Life 887 EMCCD camera (Oxford Instruments) using Micro-Manager 1.4 software (Edelstein et al., 2014). The acquisition of Super-Resolution Radial Fluctuations (SRRF) images (Gustafsson et al., 2016; Lee et al., 2018) was performed with an electron-multiplying gain of 200 and an exposure time of 10 ms, 30 frames per image, producing a time resolution of ∼1 s per SRRF image.

The 16-bit SRRF image sequences of live retina slices were processed through Fiji and MATLAB R2018a (MathWorks; RRID:SCR_001622) software to identify local fluorescence intensity peaks labeled as ROIs and to capture ROI intensity through time. ROIs were initially identified manually in maximum intensity projections of the image sequence through time, which permitted identification of Cx36-GCaMP gap junction plaques despite fluctuations of fluorescence intensity and also captured any small movements of the slice during the acquisition. Fluorescence intensity within each selected ROI was extracted from each image using MATLAB. Subsequent analyses used R software (R Project for Statistical Computing, www.r-project.org; RRID:SCR_001905). For each ROI, the baseline decay in fluorescence intensity (F_0_) with time was estimated with the whole SRRF image stack excluding images acquired during the puff and the subsequent 20 s responding window. By fitting the baseline with an exponential decay model (Eq. 1), the difference (ΔF_t_) between the measured fluorescence levels and the baseline (F_0,t_) was then divided by the corresponding baseline fluorescence level to calculate response at each time point (ΔF/F_0_). Response area under the curve for each ROI was calculated as the sum of response values from the puff through the end of the acquisition (Eq. 2) as follows:

(1) $$\text{Y }=\;\left({\text{Y}}_{0}-\text{ plateau}\right)\;*{\text{ e}}^{-\text{X}/\lambda }+\text{ plateau,}$$

where Y_0_ denotes the initial intensity of GCaMP fluorescence, 1/λ denotes the rate of decay, and plateau denotes the late residual intensity of decaying GCaMP fluorescence; and

(2) $$\text{AUC }=\sum \;({\text{δF}}_{\text{t}}∕{\text{ F}}_{0,\text{t}}).$$

Positive GCaMP responses to different puffing conditions were defined by at least three points of measurements in a 20 s postpuff responding time window (between the 11th and the 30th s of live imaging) exhibiting ΔF larger than two times the SD of measurements acquired outside the responding window used to calculate the baseline. The fraction of gap junctions responding was calculated as a population measure within each slice experiment as the number of individual gap junction plaques with a positive response, as defined above, divided by the total number of gap junction plaques analyzed in that experiment.

### Statistical analyses

Statistical comparisons were performed using estimation statistics (Ho et al., 2019) using Web-based tools accessible at https://www.estimationstats.com/#/. Additional traditional statistical analyses were performed using Prism software (GraphPad; RRID:SCR_002798).

## Results

### Development of transgenic mice to monitor electrical-synapse-localized calcium signaling

**Figure 1.**
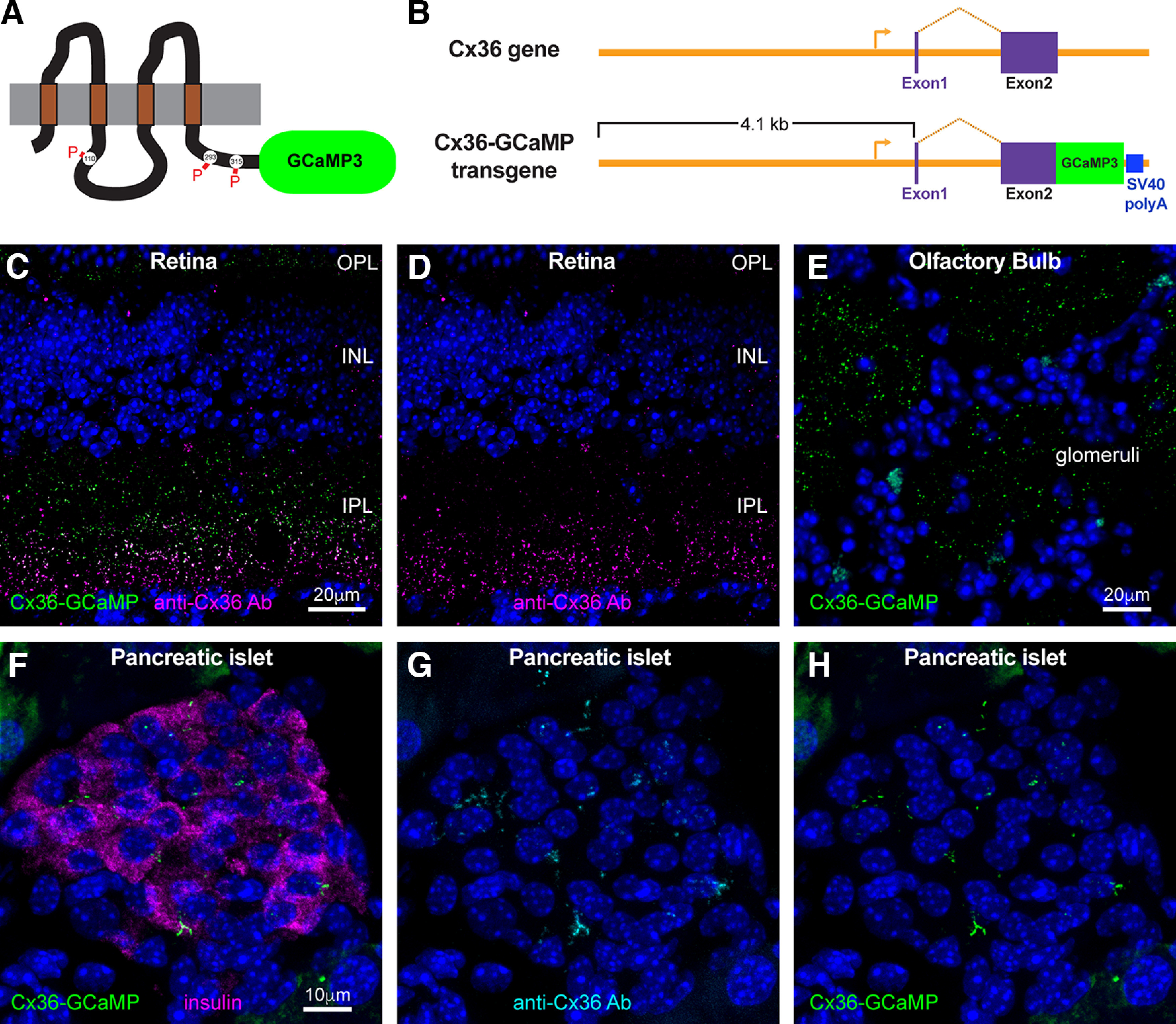
Cx36-GCaMP transgenic mouse. ***A***, Cx36-GCaMP consists of mouse Cx36 with GCaMP3 fused to the C terminal. Regulatory phosphorylation sites of Cx36 are shown. Cx36-GCaMP expressed in cultured cells has been shown to display normal functional regulation by protein kinases (Moore et al., 2020). ***B***, The Cx36-GCaMP transgene consists of 4.1 kb of genomic sequence upstream of mouse Cx36 exon 1, exon 1, intron 1, and exon 2 and SV40 polyadenylation signal derived from the Cx36-GCaMP expression plasmid. The endogenous transcription initiation site is indicated by the right arrow, and transcript splicing is indicated by the chevron between exons 1 and 2. ***C***, ***D***, Cx36-GCaMP expression in retina of transgenic mice. Cx36-GCaMP labeled with an anti-GFP antibody is green; Cx36 immunolabeling is magenta. Cx36-GCaMP is expressed in punctate spots in both IPL and OPL, where Cx36 is normally expressed. INL, Inner nuclear layer. Scale bars: 20 μm. ***E***, Cx36-GCaMP expression in olfactory bulb glomeruli labeled with anti-GFP antibody. Scale bar: 20 μm. ***F–H***, Expression of Cx36-GCaMP in the pancreas. Cx36-GCaMP (green) is found between islet cells in the pancreas that contain insulin (***F***, magenta). Labeling with an anti-Cx36 antibody (***G***, cyan) is nearly identical to labeling of Cx36-GCaMP with an anti-GFP antibody (***H***, green). Scale bars: 10 μm.

Calcium signaling has been demonstrated to instigate profound changes in coupling of Cx36-containing electrical synapses (Pereda et al., 1998; Kothmann et al., 2012; Turecek et al., 2014; Sevetson et al., 2017), but to date it has not been possible to monitor changes in the calcium microenvironment surrounding electrical synapses. This presents challenges in understanding how neuronal activity may influence functional connectivity of electrically coupled networks. To overcome this problem, we used the gap junction–tethered calcium biosensor Connexin36-GCaMP (Cx36-GCaMP; Moore et al., 2020) to develop transgenic mice with electrical synapses tagged with the calcium biosensor. Cx36-GCaMP contains GCaMP3 (Tian et al., 2009) fused to the C terminus of mouse Cx36 (Fig. 1*A*). This fusion construct reports the local calcium microenvironment around gap junctions and retains both channel function and functional plasticity (Moore et al., 2020). We developed a transgene construct containing ∼5 kb of the mouse *Cx36* (*gjd2*) gene, including ∼4 kb of the upstream regulatory region, exon 1 and intron 1, with exon 2 derived from Cx36-GCaMP (Fig. 1*B*). This construct was used to develop transgenic mice (see above, Materials and Methods), and F1 offspring were screened for uniformity of expression in the retina. Two lines with consistent expression, G01 and G43, have been propagated by incrossing. Data from G01 are shown in this communication.

Figure 1*C* shows expression of Cx36-GCaMP in vertical sections of mouse retina with signal enhanced with an anti-GFP antibody (green). Cx36-GCaMP was expressed in punctate clusters in the retinal IPL and outer plexiform layers (OPL), a distribution typical of Cx36 expression in gap junctions in the retina (Feigenspan et al., 2001; Mills et al., 2001). Immunostaining with anti-Cx36 antibody revealed precisely overlapping distribution of the two labels through most of the inner plexiform layer (Fig. 1*C*,*D*). A narrow band of Cx36 gap junctions in the lower edge of the inner plexiform layer failed to contain Cx36-GCaMP, suggesting that at least one cell type did not express the transgene. Ninety-six ± 3% of Cx36-GCaMP puncta colocalized with anti-Cx36 immunostaining, whereas 75 ± 10% of Cx36 puncta colocalized with Cx36-GCaMP (*n* = 8 animals).

Cx36-GCaMP was also found in a punctate distribution in other brain regions known to express Cx36. As an example, Figure 1*E* shows labeling for Cx36-GCaMP in olfactory bulb glomeruli, where Cx36 expression is enriched (Belluardo et al., 2000; Degen et al., 2004), and Cx36 supports lateral excitation and spike synchrony of mitral cells (Christie et al., 2005; Christie and Westbrook, 2006). We also examined expression of Cx36 in the pancreas, where Cx36 is expressed in endocrine beta cells and is required for synchrony of activity and efficient secretion of insulin (Calabrese et al., 2001; Serre-Beinier et al., 2009). Figure 1*F* shows that Cx36-GCaMP is found between insulin-containing beta cells of pancreatic islets. Furthermore, Cx36-GCaMP labeling overlaps completely with labeling for Cx36 (Fig. 1*G*,*H*), which includes both endogenous Cx36 and transgenic Cx36-GCaMP.

In mammalian retina, the largest Cx36 gap junctions are found predominantly in the AII amacrine (amacrine type 2) cells (Feigenspan et al., 2001; Mills et al., 2001) and are located in the lowest sublaminae of the IPL. As much as 98% of Cx36 is localized to AII amacrine cells in this lowest layer (Mills et al., 2001). This is the area where Cx36-GCaMP expression is missing from some large gap junctions (Fig. 1*C*), suggesting that AII amacrine cells may not express the transgene in this transgenic line. Some Cx36-GCaMP expression was detected in the lowest layer (Fig. 1*C*). AII amacrine cells make gap junctions both with each other and with several classes of cone On bipolar cells (Kolb and Nelson, 1983; Anderson et al., 2011; Sigulinsky et al., 2020), and trafficking to these two classes of gap junctions is dependent on different factors (Meyer et al., 2014). One potential explanation for the Cx36-GCaMP signal in the lower IPL is that it is expressed in some cone On bipolar cells and localized to gap junctions with AII amacrine cells. This hypothesis will require further investigation to verify.

### The calcium microenvironment is dynamic at electrical synapses

**Figure 2.**
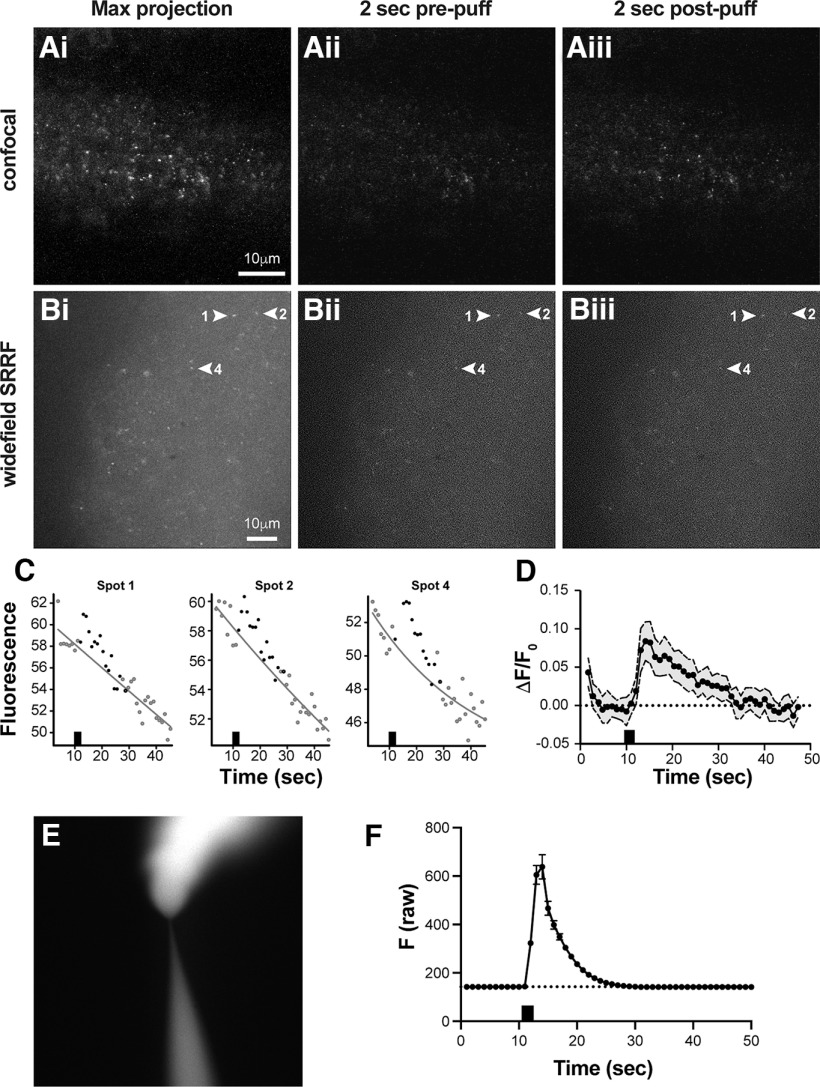
Glutamate puff responses of Cx36-GCaMP in retina slices. ***Ai–iii***, Confocal imaging of a retina slice preparation centered on the inner plexiform layer presented with a 1 s puff of 750 μm glutamate (***Ai***). Maximum intensity projection across the full 20 s time course (***Aii***). Single image 2 s before glutamate puff (***Aiii***). Single image 2 s after glutamate puff. ***Bi–iii***, Widefield SRRF imaging of retina slice preparation centered on the inner plexiform layer presented with a 2 s puff of 1 mm glutamate (***Bi***). Maximum intensity projection across the full 45 s time course (***Bii***). Single image 2 s before glutamate puff (***Biii***). Single image 2 s after glutamate puff. Scale bars in ***A, B***: 10 μm. ***C***, Raw fluorescence intensity sequences of three example gap junctions from the sequence in ***Bi*** (arrowheads). The glutamate puff is indicated by the black box on the time axis. Black spots following the puff are data points within the designated responding window used to assess a positive response. Gray lines are the F_0_ baselines fit to the intensity data outside the responding window (gray data points). ***D***, Mean responses of 15 gap junctions from the sequence in ***Bi–iii*** expressed as ΔF/F_0_. Error bars are ± 1 SD. The glutamate puff is shown by the black box on the time axis. ***E***, ***F***. Time course of fluorescence of a Lucifer yellow dye puff in the recording chamber under the superfusion conditions of experiments shown in this study. ***E***, Image of a puffing pipette and dye plume at the mouth of the pipette. ***F***, Mean (symbols) ± 1 SD of raw fluorescence intensities of 12 spots within the plume at a greater distance from the pipette over the course of one puff; 2 s puff is indicated by the black box on the *x*-axis.

AII amacrine cell gap junctions display profound activity-dependent potentiation driven by activation of NMDA receptors and subsequent activation of calcium/calmodulin-dependent protein kinase II (CaMKII; Kothmann et al., 2012). This signaling pathway appears to require calcium influx, but the existence of a glutamate-driven calcium signal at electrical synapses has not been demonstrated. To examine this signaling pathway, we imaged slice preparations from Cx36-GCaMP mouse retina presented with puffs of glutamate; 1 mm glycine was present in both superfusion and puff solutions to enable NMDA receptor activation by glutamate. We used two types of apparatus for these experiments, a confocal microscope using scanning laser excitation ((Fig. 2*Ai–Aiii*) and a widefield microscope using SRRF imaging (Gustafsson et al., 2016; Lee et al., 2018; Fig. 2*Bi–Biii*; see above, Materials and Methods). Both systems use visible light excitation, so retinal On pathway light responses were suppressed by the presence of l-AP4 in perfusion and puff solutions throughout the experiments. Figure 2, *Ai* and *Bi*, show maximum intensity projections through time of a 20 s confocal sequence and a 45 s SRRF sequence, respectively, centered on the retinal inner plexiform layer where amacrine cell, ganglion cell, and some bipolar cell gap junctions are located. Individual frames are shown 2 s before a 2 s glutamate puff (Fig. 2*Aii*,*Bii*) and 2 s after the puff (Fig. 2*Aiii*,*Biii*). Individual Cx36-GCaMP gap junctions can be followed through time in these experiments (Movie 1).

Movie 1. Cx36-GCaMP mouse retina slice recording. Response of a slice to a 2 s puff of 1 mm glutamate begun at 4 s; 20 s total record time. 10.1523/ENEURO.0493-22.2023.video.1

Figure 2*C* shows raw fluorescence intensity of three individual Cx36-GCaMP gap junctions from the sequence in Figure 2*B* (arrowheads). A single exponential baseline was fit to each individual spot to represent F_0_, as shown by the gray lines in Figure 2*C*. In each spot, an increase in fluorescence time locked to the application of the glutamate puff (black bar) was detected. This was superimposed on a declining baseline that was at least partially because of a tissue-wide increase in fluorescence caused by the onset of the imaging light and is calculated into the F_0_ baseline. In the experiment in Figure 2*B* 15 of 15 measured spots showed positive responses (at least three time points 2 SD above the baseline); 18 of 34 measured spots showed positive responses in the experiment in Figure 2*A*. These spots showing positive responses are interpreted to be gap junctions that experienced a transient increase in local calcium concentration in response to the glutamate puff. Spots lacking a response are interpreted to be gap junctions that did not encounter a change in local calcium concentration above the noise level of the measurement time locked to the puff stimulus. Figure 2*D* shows the average glutamate puff response ± 1 SD of the 15 measured Cx36-GCaMP spots from the experiment in Figure 2*B*. Calcium rose rapidly at the gap junctions immediately following the glutamate puff and declined steadily as the puff washed out. Control experiments using puffs of Lucifer yellow dye revealed persistence of the puff solution was similar to the duration of Cx36-GCaMP responses (Fig. 2*E*,*F*), suggesting that the duration of Cx36-GCaMP calcium responses in these experiments depended on the washout of glutamate from the tissue slice. It is unclear to what extent Cx36-GCaMP responses may be shaped by cellular factors such as the kinetics of ion channels or transporters. These experiments indicate that a large fraction of electrical synapses expressing Cx36 GCaMP experience dynamic increases in calcium on glutamate stimulation.

### Calcium transients come from multiple sources

**Figure 3.**
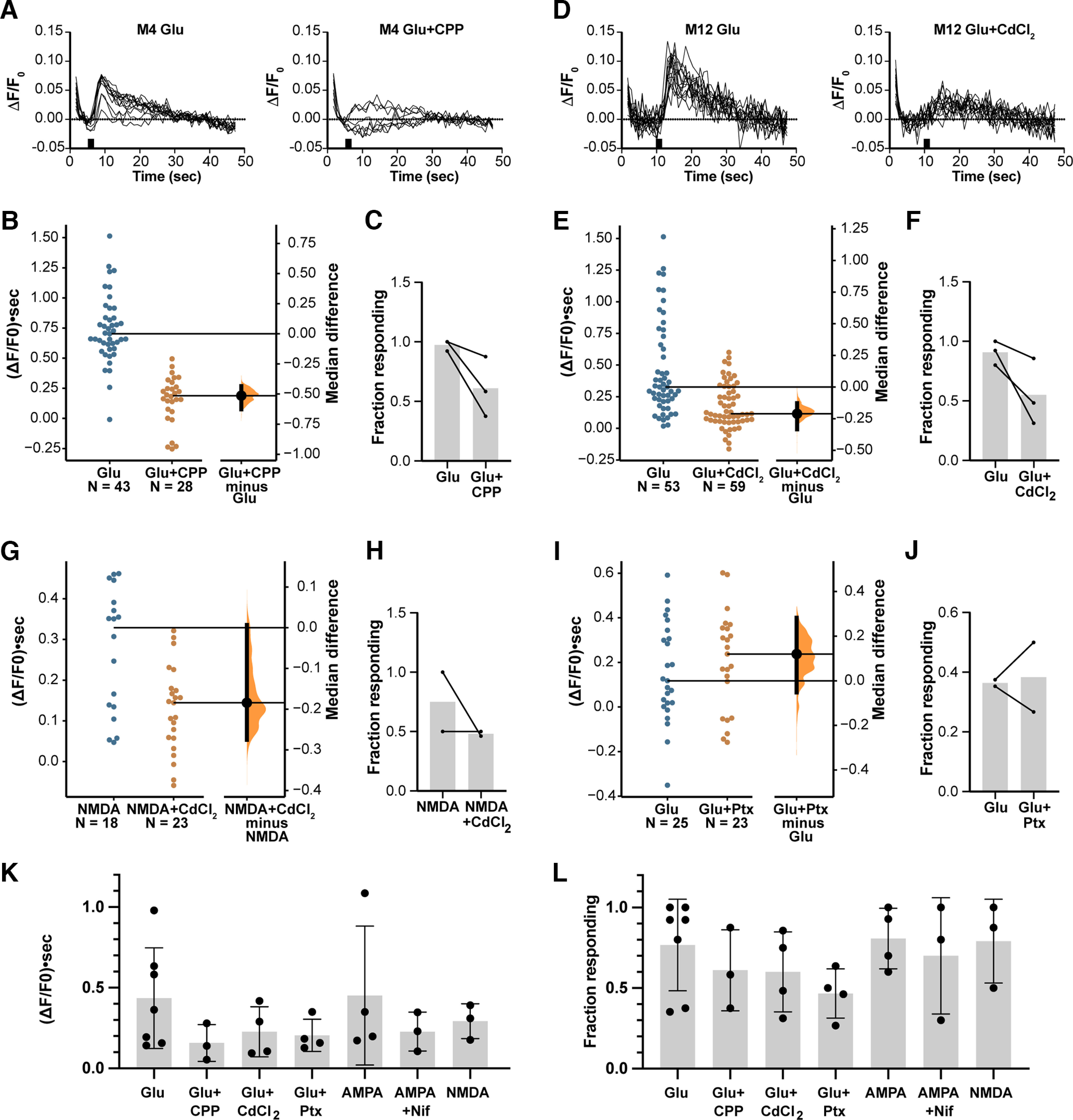
Pharmacological analysis of sources of calcium at Cx36-GCaMP electrical synapses. ***A***, Left, Individual responses to a 2 s glutamate puff (black bar) of 13 Cx36-GCaMP spots. There is some variation in amplitude, with some spots failing to respond. Right, Individual responses of eight Cx36-GCaMP spots in the same slice with 10 μm CPP in the bath and puff solutions. Very few spots showed responses, but some persisted. ***B***, Gardner–Altman estimation plot of pooled individual spot response amplitudes from three slices recorded in control and CPP conditions. Median responses are indicated by the black lines. Right, The frequency distribution of the median treatment, control difference in 5000 bootstrap replicates; 95% confidence intervals of the median difference are indicated by the black lines. ***C***, Fraction of Cx36-GCaMP spots showing a positive response in the three slices shown in ***B***. ***D***, Individual responses to a glutamate puff of 15 spots in control (left) or solutions containing 100 μm CdCl_2_ (right) in the same slice. ***E***, Gardner–Altman estimation plot of pooled individual spot response amplitudes from three slices recorded in control and CdCl_2_ conditions. ***F***, Fraction of Cx36-GCaMP spots showing a positive response in the three slices shown in ***E***. ***G***, Gardner–Altman estimation plot of pooled individual spot response amplitudes to puffs of 10 μm NMDA from two slices recorded either in control solution or with 100 μm CdCl_2_ in the perfusion and puff solutions. ***H***, Fraction of Cx36-GCaMP spots showing a positive response in the two slices shown in ***G***. ***I***, Gardner–Altman estimation plot of pooled individual spot response amplitudes to puffs of glutamate from two slices recorded either in control solution or with 100 μm Picrotoxin in the perfusion and puff solutions. ***J***, Fraction of Cx36-GCaMP spots showing a positive response in the two slices shown in ***I***. ***K***, Population average responses to puffs of 1 mm glutamate, 100 μm AMPA, or 10 μm NMDA. Response amplitudes are shown in ***K*** and fraction of gap junctions responding in ***L***. Each point represents average responses from Cx36-GCaMP spots in one slice; bars indicate the mean, and error bars indicate 1 SD. A total of 16 slices from 12 animals is included.

Direct modulation of gap junction coupling by activity and glutamate has been shown to depend on NMDA receptors in the AII amacrine cells (Kothmann et al., 2012). To examine the potential sources of glutamate-induced calcium signals at the broader sample of Cx36-GCaMP gap junctions in our experiments, we performed a series of pharmacological interventions. Puffs of glutamate produced responses of variable amplitude in different individual gap junctions (Fig. 3*A*, left) and between slices. This suggests that individual gap junctions within the slice encounter quantitatively different changes in local calcium concentration, and some reveal no detectable change. Note that because of the wide-field imaging paradigm, some background tissue fluorescence is incorporated into the F_0_ baseline used to calculate ΔF/F_0_. So, to the extent that scattered light from positive responses is incorporated into F_0_, the fraction subtracted from positive spot responses may affect the response amplitude measured for small gap junctions more than that measured for larger ones exposed to comparable calcium changes. Glutamate puffs produced a population-wide average time-integrated response of 0.436 ± 0.312 (ΔF/F_0_) • sec (*n* = 7 slices from 7 animals), with 77 ± 28% (*n* = 7) of gap junctions showing a positive response (Fig. 3*K*,*L*). Inhibition of NMDA receptors with CPP largely blocked responses of some gap junctions, whereas other gap junctions retained some response (Fig. 3*A*, right), indicating that some of the gap junctions imaged required NMDA receptors for the local calcium rise, although not all did. Figure 3*B* shows individual spot responses, and Figure 3, *C* and *L*, shows the fraction of spots showing a positive response in three slices from three animals in which both control glutamate puff and glutamate and CPP conditions were tested. The median difference of the response area was −0.514 (ΔF/F_0_) • sec, and the *p* value of the two-sided permutation *t* test of 5000 bootstrap samples of the data was 0.0. This indicates than NMDA receptors were required for a large fraction of transient calcium signals in response to glutamate puffs detected at Cx36-GCaMP gap junctions in the inner plexiform layer.

Suppression of glutamate-driven calcium signals at Cx36 gap junctions by an NMDA receptor antagonist was the expected result. Nonsynaptic NMDA receptors very closely associated with Cx36 gap junctions have been shown to contribute significantly to the signaling that drives CaMKII phosphorylation of Cx36 in AII amacrine cells (Kothmann et al., 2012). However, as described above, it is possible that Cx36-GCaMP is not expressed in AII amacrine cells, and it is known that Cx36 is expressed by additional cell types in the inner retina, including retinal ganglion cells and other amacrine cells (Hidaka et al., 2002; Schubert et al., 2005; Hoshi and Mills, 2009; Pan et al., 2010; Kirkby and Feller, 2013; Harrison et al., 2021). Furthermore, most ganglion cells and some amacrine cells use NMDA receptors in postsynaptic response to glutamatergic bipolar cells (Massey and Miller, 1990; Marc, 1999; Gustafson et al., 2007). In cells with synaptic NMDA receptor responses, depolarization may activate voltage-gated calcium channels, providing another potential source of calcium at Cx36 gap junctions. To examine whether voltage-gated calcium channels were involved, we treated retina slices with CdCl_2_ to block calcium channels nonselectively. Figure 3*D* shows individual spot responses from one slice to a puff of glutamate without or with 100 μm CdCl_2_. CdCl_2_ reduced the amplitude of most spot responses. Figure 3*E* shows individual spot responses, and Figure 3*F* shows the fraction of spots showing a positive response in three slices from three animals in which both control glutamate puff and glutamate plus CdCl_2_ conditions were tested. The median difference of the response area was −0.211 (ΔF/F_0_) • sec, and the *p* value of the two-sided permutation *t* test of 5000 bootstrap samples of the data was 0.0002. Population average data from four slices are shown in Figure 3, *K* and *L*.

These results suggest that voltage-gated calcium channels contributed to the calcium responses of the population of electrical synapses in these slices. However, it has been reported that CdCl_2_ directly inhibits NMDA receptors (Legendre and Westbrook, 1990; Tu et al., 2016), which confounds this interpretation. As a more focused test of the involvement of voltage-gated calcium channels, we used NMDA puffs to stimulate NMDA receptors more specifically. This should activate both synaptic NMDA receptor populations, which may depend on voltage-gated calcium channels to generate calcium transients at distant gap junctions, and nonsynaptic NMDA receptor populations that may not if they are close enough to Cx36 gap junctions to elicit a calcium signal via direct calcium influx through the NMDA receptor channel. Figure 3*G* shows that in two slices from two animals, NMDA puffs in the presence of CdCl_2_ tended to elicit smaller responses than control NMDA puffs. The median difference of the time-integrated response area was −0.184 (ΔF/F_0_) • sec, and the *p* value of the two-sided permutation *t* test of 5000 bootstrap samples of the data was 0.0016. There was a variable effect on the fraction of gap junctions showing a positive response to NMDA puffs on treatment with CdCl_2_ (Fig. 3*H*). These modest effects of CdCl_2_ on response amplitude and fraction of gap junctions displaying a response indicate that CdCl_2_ did not fully inhibit NMDA receptors at the concentration applied. The much larger effect on glutamate-stimulated responses suggest that voltage-gated calcium channels supplemented the transient calcium increase at Cx36 gap junctions when all types of glutamate receptors were activated. Finally, we examined the effects of the selective l-type calcium channel blocker nifedipine (20 μm) in experiments with puffs of 100 μm AMPA to activate AMPA receptors without substantial contributions from NMDA receptors. In this paradigm, nifedipine tended to reduce the amplitude of the population average response (Fig. 3*K*,*L*), again suggesting a contribution of voltage-gated calcium channels to the response.

As a final test, we also evaluated the effects of blocking ionotropic GABA receptors, which are likely to be activated in the glutamate puff paradigm because of glutamate excitation of amacrine cells and synaptic GABA release in the IPL. Figure 3*I* shows that application of picrotoxin had no effect on the time-integrated response area [median difference 0.12 (ΔF/F_0_) • sec; *p* value of the two-sided permutation *t* test of 5000 bootstrap samples of the data was 0.224), and Figure 3*J* shows that there was no consistent effect on the fraction of gap junctions showing a positive response in two slices from two animals. Thus, GABA receptor activation appeared to have little influence on calcium transients at Cx36-GCaMP gap junctions at the population level.

## Discussion

### Activity-dependent tuning of electrical coupling

**Figure 4.**
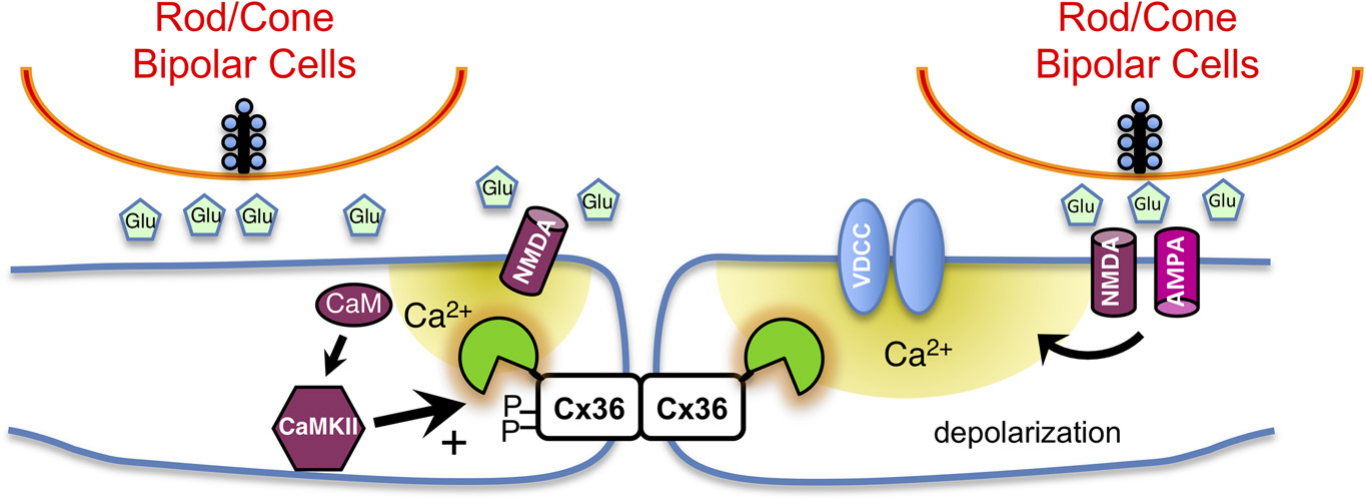
Schematic model of sources of calcium available for electrical synapse plasticity. Left, A Ca^2+^–CaMKII signaling pathway has been well documented to enhance Cx36 coupling by promoting Cx36 phosphorylation; an opposing mechanism activating calcineurin and dephosphorylating Cx36 has also been proposed. Extracellular sources of calcium triggered by glutamatergic synaptic activity are shown, including direct influx through NMDA receptors (left) and indirect influx through voltage-gated calcium channels triggered by membrane depolarization (right). Direct calcium influx may involve nonsynaptic NMDA receptors as shown, or may involve synaptic NMDA or calcium-permeable AMPA receptors in close proximity to electrical synapses. Indirect signaling may involve synapses at any distance from the electrical synapse and may also occur in excitatory neurons shown as presynaptic in this diagram.

Electrical synapses are abundant in the retina, being found in all classes of retinal neuron and being subject to extensive plasticity (Ribelayga and O'Brien, 2017; O'Brien and Bloomfield, 2018). Activity-dependent plasticity is a subset of the types of plasticity observed at electrical synapses. In retinal AII amacrine cells, light-dependent and bipolar cell activity–dependent signaling drives a more than threefold increase in coupling, as measured by the diffusion coefficient for Neurobiotin tracer diffusion through the AII amacrine cell network (Kothmann et al., 2012). This signaling is initiated by spillover glutamate-activating nonsynaptic NMDA receptors, with activation of CaMKII and phosphorylation of Cx36 (Kothmann et al., 2012; Fig. 4, left). In the present study, we find that NMDA-receptor-dependent calcium transients are widespread at Cx36 gap junctions in the retina. However, direct calcium flux through NMDA receptors is not the only source of calcium that may induce Cx36 plasticity in the retinal inner plexiform layer. Additional signaling driven by glutamate-dependent activation of voltage-gated calcium channels (Fig. 4, right) contributes to calcium transients that we can detect at Cx36-GCaMP gap junctions. Although not unexpected, the presence of calcium fluctuations at retinal electrical synapses derived from voltage-gated calcium channels has not been formally demonstrated. This expands the repertoire of signaling mechanisms that have potential to regulate electrical synapse plasticity.

The most striking observation of this study is that >90% of gap junctions containing Cx36-GCaMP experience transient increases in calcium on glutamate puff stimulation. These gap junctions have been selected essentially randomly from the entire population of Cx36 gap junctions in the retinal inner plexiform layer that harbor Cx36-GCaMP. With the caveat that AII amacrine cells may not express Cx36-GCaMP, this observation is all the more striking, suggesting that a dynamic calcium microenvironment around electrical synapses is commonplace in retinal neurons.

Studies of phosphorylation of Cx36 in the retina demonstrate that gap junctions within one micron of each other on the same dendrite can be in very different phosphorylation states and, hence, different states of functional plasticity (Kothmann et al., 2009). This emphasizes the fact that functional plasticity of each individual Cx36 gap junction plaque is highly dependent on local cellular signaling. Most studies of calcium dynamics in neurons record signals in the cell soma, often at a great distance from electrical synapses. Although such changes may be correlated with calcium changes in the vicinity of electrical synapses, it has not previously been possible to assess whether that is the case. Cx36-GCaMP allows the direct investigation of calcium signals relevant to the functional regulation of Cx36 electrical synapses.

Cx36 is intrinsically capable of plasticity over an order of magnitude dynamic range (O'Brien, 2019). Outside the retina, long-term potentiation of electrical synapses is driven by calcium influx through synaptic NMDA receptors in Mauthner cell mixed synapses (Pereda and Faber, 1996; Haas et al., 2016) and through nonsynaptic NMDA receptors in inferior olive neurons (Turecek et al., 2014). Curiously, synaptic activation of NMDA receptors in inferior olive neurons has the opposing action to trigger electrical synaptic depression (Mathy et al., 2014). This depression is similar to the effects of activity in thalamic reticular neurons (Landisman and Connors, 2005; Haas et al., 2011). In the latter, two distinct pathways cause electrical synaptic depression; a calcium-independent pathway depends on presynaptic glutamate activation of metabotropic glutamate receptors, whereas the own activity of the neuron itself drives a calcium-dependent pathway that involves voltage-gated calcium channels and recruitment of intracellular store calcium (Sevetson et al., 2017). An activity- and calcium-dependent potentiation of electrical synapses can also be elicited in the same neurons under specific conditions that limit calcium influx, indicating that calcium drives bidirectional plasticity dependent on the specific type of activity and level of calcium experienced (Fricker et al., 2021). The similarities and differences in signaling in these example networks demonstrate that the signaling pathways that control electrical synapse plasticity are circuit specific and very sensitive to the magnitude of the calcium signal experienced locally at the electrical synapse.

Retinal ganglion cells adapt in a matter of seconds to both average image contrast and to the local distribution of contrast features (Smirnakis et al., 1997). Our experiments in the Cx36-GCaMP mouse demonstrate that a large fraction of electrical synapses experience a dynamic calcium microenvironment, providing the molecular underpinnings to contribute to adaptation on a seconds time scale. This mouse will be a useful tool to examine the plasticity within specific circuits when paired with strategies to label specific cell types.

## Synthesis

Reviewing Editor: Michael Michaelides, NIDA-NIH Decisions are customarily a result of the Reviewing Editor and the peer reviewers coming together and discussing their recommendations until a consensus is reached. When revisions are invited, a fact-based synthesis statement explaining their decision and outlining what is needed to prepare a revision will be listed below. The following reviewer(s) agreed to reveal their identity: Julie Haas, Matthew Van Hook.

Reviewer 1

The main goal of this ms is to present a new tool to the community: a cx36-GCaMP3 mouse, with which one can visualize and measure calcium transients that occur in close proximity to electrical synapses. The motivation and use for this tool is to better identify the sources and effectors of calcium that are associated with plasticity of cx36-based electrical synapses, which have been recently demonstrated in several neuronal systems. Authors present confocal and time-lapsed fluorescent images in retinal tissue from the transgenic mice. These data very nicely demonstrate colocalization of the fusion construct with cx36 stained by the standard antibody, and flux of calcium by the sensor with the expected amplitude and dynamics. Pharmacological experiments are added to time-lapse imaging, which reveal that both NMDA and VGCC channels are sources of calcium transients.

Overall, this is an exciting new tool that should be of wide interest, the data seem sound and well presented, and the paper is well written.

Major:

As the main goal is to introduce this tool to the community, it would be nice to present images from other major areas and nuclei known to express cx36.

Fraction responding (Fig. C, F, H, J): I’m unsure of what is communicated here, either numerically or rhetorically. Do the authors distinguish between weak spots that fall below detection threshold after drug reduction, and spots that truly stop responding? Limiting the analysis to only spots with a response initially 2x (or more) above detection threshold would be one way to distinguish between those possible outcomes. Also, I’m not sure of how the fraction responding is interpreted by the authors - does it imply that GJ channels or plaques are segregated (ie some near a specific calcium channel, others not)? It is unremarked-on in the manuscript. N, taken as the number of slices, at 2-3 seems too low in some cases to make a distinction.

The results are presented in text, but it would be nice to add colocalization numbers in bar plot or visual form to Fig. 1.

Minor:

In Fig. 1, cx36 seems remarkably and unexpectedly absent from glomeruli (cf Christie et al 2005, Zhang 2003), unless I’m misreading the label. Is this true, and consistent?

Are there limitations that directed the choice for GCaMP3, or can one expect that newer and faster GCaMPs can be used in similar manner?

In terms of preserved function, the authors cite Moore et al. who show that dye coupling between HeLa cells is maintained and regulated normally. It would be a nice addition to reconfirm that electrical synapse function is preserved in some form in the present system.

Will the mice be publicly available?

Regarding calcium and plasticity, citations should be added to Fricker et al. (2021), and Welzel and Schuster (2018) which is in innexin but suggests calcium sensitivity is preserved.

Reviewer 2

This manuscript describes the development of a transgenic mouse expressing a fusion protein of connexin-36 (major gap junction protein in the cns) and GCaMP6 (a fluorescent Ca2+ sensor) and its implementation in probing the origins of Ca2+ signals at gap junctions in the inner plexiform layer of the retina. Overall, the mouse will likely be a useful tool for future work. The data probing Ca2+ at inner retinal gap junctions is interesting. This is potentially helpful in understanding the mechanisms driving Ca2+-dependent dynamic regulation of gap junction conductance in the inner retina, which contributes to adaptation, allowing retinal circuitry to dynamically respond over 10 log units of light intensity. I have concerns about statistical approaches as well as about the interpretation of Cd++ data and think that the conclusion that VGCCs play a role needs more support than provided in the current manuscript. I have the following suggestions for improving the manuscript:

1) I believe the supplemental figures should be incorporated into the main manuscript and not a supplement.

2) It appears that individual cx36-GCaMP puncta are treated as independent for the purposes of statistical testing. It is unclear whether statistics approaches account for the non-independence of each punctum in response to single puffs or drug applications. Relatedly, an N=2 slices (Fig 3 G-J) might be too small of a sample for drawing any conclusions.

3) The authors should clarify their interpretation on whether the Ca2+ signals they’re measuring represent local calcium concentration changes at the gap junctions OR whether they might be global Ca2+ changes that are simply detected at the gap junctions (due to localization of the sensor rather than localization of the Ca2+ itself). The authors seem to lead toward the former interpretation based on the way the data are described (and based on the Fig 4 schematic), but I suspect the latter is actually the scenario.

4) In terms of functional relevance, AII gap junctions have received the most attention in the literature given the roles of AII gap junctions in dim-light encoding and Ca-dependent regulation. However, the authors suggest that AII gap junctions might not have the Cx36-GCaMP expression based on the pattern of labeling in the IPL. This is surprising and I think that the manuscript and its interpretation could be strengthened with some data speaking to which retinal cell types express the Cx36-GCaMP.

5. The statement that the LY puff fluorescence time course (line 292 and Fig S2) is similar to the Ca2+ signal time course does not seem to be true. Rather, the Ca2+ signals seem to take near 10x as long to decline to baseline. This statement and interpretation of the LY puff need to be revised. A glutamate puff will likely result in substantial AMPA receptor desensitization.

6) Lines 339-342: it is not clear why synaptic vs non-synaptic NMDA receptors would be expected to have dependence on VGCCs. The logic of this statement is not clear.

7) The interpretation of Cd2+ effects as evidence for VGCC contributions needs further clarification. In 3H, it’s not clear how the Cd effect points to a contribution of VGCCs when the Cd might just be inhibiting NMDARs. The conclusion might be strengthened with Ca-channel specific blockers. For instance, nifedipine is listed in the methods section, but it’s not apparent that data with nifedipine is included in the manuscript. L-type channels seem likely candidate (and would be mostly blocked by nifedipine), but it’s not clear why data with nifedipine were not included.

## Author Response

Responses to reviewers:

We greatly appreciate the thoughtful critiques of the two reviewers of this manuscript. We have revised the manuscript in accordance with the comments provided. In the revised manuscript provided for review, new text is highlighted in blue font. Responses to each of the reviewers’ comments are provided below. Changes to the manuscript are described in detail in these responses.

Reviewer #1

1. It would be nice to present images from other major areas and nuclei known to express cx36.

We are primarily a retina lab, so we have focused our efforts on the retina. Unfortunately, this strain of mice was lost when the lab changed institutions in 2021. We have initiated a project to rederive the line from cryopreserved sperm, but this will take many months. For the purposes of this resubmission, we have added other immunostaining that we performed earlier on the pancreas, which expresses Cx36 in islet cells. These also express Cx36-GCaMP and are shown now in figure 1 F-H. We have also modified the color space in the immunostaining in figure 1 to be more color-blind friendly, replacing red with magenta in 3 images.

2. Fraction responding (Fig. C, F, H, J): I’m unsure of what is communicated here, either numerically or rhetorically. Do the authors distinguish between weak spots that fall below detection threshold after drug reduction, and spots that truly stop responding? Limiting the analysis to only spots with a response initially 2x (or more) above detection threshold would be one way to distinguish between those possible outcomes.

These are good points. The gap junctions measured are initially identified by making a maximum intensity projection (MIP) through time and selecting local maxima as gap junction plaques to measure. We hadn’t stated this in the Methods, so it has now been added in lines 235-239. This method is agnostic to the response of the gap junction during the experiment, except that a particularly dim plaque (i.e. small) that never responded might be bypassed in selection of local maxima. This method is necessary because plaques are indeed small and imaging is noisy, so it is possible to miss many gap junctions in any given frame of the acquisition. This local maximum method does not miss gap junctions of some minimum size that do not respond because GCaMP3 has some intrinsic fluorescence, making it detectable at baseline. Note however that we only select a small fraction of the gap junctions in each slice (due to the intensive individual analysis) and that individual gap junction plaques assessed in a drug treatment condition are not necessarily the same ones that were assessed in the control condition for the same slice. While we did do this for some preliminary experiments, it was evident that small changes in focal depth or movement of the slice while changing solutions and puff pipettes, along with the sheer number of gap junction plaques, made it impractical to do that analysis for every slice. So these are considered population samplings.

So, to address your question, for each acquisition sequence, a group of Cx36-GCaMP plaques is selected from the MIP in ImageJ and intensity measurements for each ROI extracted in Matlab. Each of those plaques is individually analyzed with an individual baseline fit of the data outside of a predefined 20-second “responding window.” Positive responses are defined as plaques in which at least three time points were above the baseline by greater than 2 standard deviations of the difference of intensities of baseline time points from the fit baseline curve. So, for each acquisition sequence, there is a group of Cx36-GCaMP plaques for which an individual assessment of its response is made for each plaque. The “fraction responding” is the count of plaques with a positive response divided by the total number of plaques analyzed in that sequence. We have added clarification of this in the methods, lines 259-262.

3. Also, I’m not sure of how the fraction responding is interpreted by the authors - does it imply that GJ channels or plaques are segregated (ie some near a specific calcium channel, others not)? It is unremarked-on in the manuscript.

Yes, the reviewer is correct in this interpretation. This is indeed the fundamental premise of the manuscript and the motivation for developing the mouse line. The fact that this was not clear means that we have not conveyed the information very effectively. I have added some sentences in the manuscript to emphasize that plaques with positive responses experience an increase in local calcium while those that lack a response do not (or it is not measurable). E.g. lines 356-360 added to the results section. See also the response to a related question from reviewer #2, point 3.

4. N, taken as the number of slices, at 2-3 seems too low in some cases to make a distinction.

The reason that the number of slices presented in the drug treatments is low is that we limited analyses only to slices for which there was a control puff that indicated that the slice was healthy and a drug treatment puff on the same slice. The learning curve for performing these experiments was rather long, and there are many more slices for with there are viable control puffs but not a drug puff, or drug puff responses without the control that we wanted to analyze. Many of these data are included in the pooled data in supplemental figure 3 (now included in the main figure 3 as panels K and L following the suggestion of reviewer #2), for which the analysis is done at the level of the whole slice.

5. The results are presented in text, but it would be nice to add colocalization numbers in bar plot or visual form to Fig. 1.

SfN has a policy not to allow two-column bar graphs (or equivalent), so I have left these values in the text. However, I did update the calculations to use a set of images that documented the animals used. The numbers are similar, but not identical, to those in the original manuscript: 75.1 {plus minus} 10.3% of Cx36 gap junctions were GCaMP positive, while 95.5 {plus minus} 2.9% of GCaMP spots were Cx36 positive.

6. In Fig. 1, cx36 seems remarkably and unexpectedly absent from glomeruli (cf Christie et al 2005, Zhang 2003), unless I’m misreading the label. Is this true, and consistent?

You may be misreading the label. The roundish patches with all of the Cx36-GCaMP label are the glomeruli containing synapses (one centered and edges of three others in the image), which are surrounded by the DAPI-stained nuclei of the somata. We think this looks remarkably like the distribution of Cx36 detected in those studies.

7. Are there limitations that directed the choice for GCaMP3, or can one expect that newer and faster GCaMPs can be used in similar manner?

GCaMP3 was the current GCaMP available when the initial construct was made. I think GCaMP4 or 5 might have been available already when we made the transgenic mouse, but we had not tested them. The improvements in GCaMP dynamic range, particularly those leading to the GCaMP6 series, have come mostly from reduction of the fluorescence of GCaMP in the absence of calcium. For the purposes of imaging Cx36-GCaMP gap junctions, which in most cases are smaller than 1 μm in diameter, these improvements work against the investigator’s ability to detect a gap junction to study. So, in a sense the long time that it took to develop and validate the mouse was fortunate, since GCaMP3 provides adequate dynamic range and allows us to detect the gap junctions in the presence of baseline calcium.

8. In terms of preserved function, the authors cite Moore et al. who show that dye coupling between HeLa cells is maintained and regulated normally. It would be a nice addition to reconfirm that electrical synapse function is preserved in some form in the present system.

I understand the sentiment. Ours is not an electrophysiology lab, but we could certainly collaborate with an electrophysiology lab to test electrical synapse function. In the current system, the native Cx36 is still present. In order to test the function of Cx36-GCaMP as an electrical synapse, one would have to breed the transgene into a Cx36 knockout background. This has its own limitations, as Meyer et al., 2014 showed that a Cx36 transgene with a C-terminal GFP tag did not traffic to all gap junction locations on its own, but required the presence of untagged Cx36 to reach all expected gap junctions. We would predict the same for Cx36-GCaMP, which has the GCaMP attached to the C-terminus of Cx36. The primary job of Cx36-GCaMP is as a biosensor to measure local calcium dynamics. It isn’t essential that it have normal electrical synapse function to perform that task. It is only required that it be trafficked to the correct location. Given that it would already be about 6 months time frame to get the Cx36-GCaMP mouse rederived, and it might be about a year to get it bred into the Cx36 knockout background, we have opted not to attempt to validate electrophysiological properties of the construct for this paper.

9. Will the mice be publicly available?

Yes, the mice will be available to investigators. We have made a proposal to Jackson Lab to donate the strain for distribution to investigators. If this is not approved, the strain will still be available directly from us.

10. Regarding calcium and plasticity, citations should be added to Fricker et al. (2021), and Welzel and Schuster (2018) which is in innexin but suggests calcium sensitivity is preserved.

Thank you for those suggestions; these are very relevant papers. I have included these in the revised manuscript, one in the introduction and one in the discussion.

Reviewer #2

1) I believe the supplemental figures should be incorporated into the main manuscript and not a supplement.

We have moved most of the supplemental figures into the main figures. The live sequence is still in a supplement.

2) It appears that individual cx36-GCaMP puncta are treated as independent for the purposes of statistical testing. It is unclear whether statistics approaches account for the non-independence of each punctum in response to single puffs or drug applications. Relatedly, an N=2 slices (Fig 3 G-J) might be too small of a sample for drawing any conclusions.

This is a good point. We have used two strategies to make comparisons of drug treatments meaningful. First, we have made statistical assessments of response amplitudes only on slices for which we have both a viable control puff and a drug treatment puff. This means that the experiments are paired, although we have not employed a paired statistical paradigm to analyze them. This is in part due to the fact that these are population measurements, as described in the response to reviewer #1’s question 2. Second, we have employed estimation statistics in order to avoid traps associated with hypothesis testing using cohorts of data that are correlated by common responses. Estimation statistics relies on effect sizes and the uncertainty within the data, along with a post-hoc bootstrap test to illustrate the probability of producing a test population distribution by random sampling of the control population.

3) The authors should clarify their interpretation on whether the Ca2+ signals they’re measuring represent local calcium concentration changes at the gap junctions OR whether they might be global Ca2+ changes that are simply detected at the gap junctions (due to localization of the sensor rather than localization of the Ca2+ itself). The authors seem to lead toward the former interpretation based on the way the data are described (and based on the Fig 4 schematic), but I suspect the latter is actually the scenario.

This is a very good point. We had not meant to imply that the calcium changes detected occurred ONLY in the vicinity of the gap junctions. Instead, the whole foundation of developing this mouse was to reveal that/if calcium DOES indeed change in the vicinity of the gap junctions, where signaling controlling coupling is localized. Note that we know from prior studies of gap junction phosphorylation states that the functional state of each gap junction is independently controlled, even within 1 micron distance on the same dendrite of the same cell (A2 amacrine cell). This is mentioned in the introduction and the significance statement, and was a major motivation to develop this animal model. The techniques we used do not allow us to compare calcium changes at individual Cx36 gap junctions with, say, calcium changes in the cell soma or some nearby dendrite. This would be a good topic for a future study. We have added a section in the discussion (lines 492-500) that points out the potential correlation or lack of correlation between global calcium changes (i.e. those measured at the soma in most studies) and those at the electrical synapses.

4) In terms of functional relevance, AII gap junctions have received the most attention in the literature given the roles of AII gap junctions in dim-light encoding and Ca-dependent regulation. However, the authors suggest that AII gap junctions might not have the Cx36-GCaMP expression based on the pattern of labeling in the IPL. This is surprising and I think that the manuscript and its interpretation could be strengthened with some data speaking to which retinal cell types express the Cx36-GCaMP.

It is true that it would be desirable to know more about which cell types in the retina express the transgene. Unfortunately, as stated in response to Reviewer #1’s question 1, this line was lost when the PI changed institutions in 2021. It will still take another 6 months for us to rederive the line and have animals to work with, so this endeavor will have to wait for a future study.

5. The statement that the LY puff fluorescence time course (line 292 and Fig S2) is similar to the Ca2+ signal time course does not seem to be true. Rather, the Ca2+ signals seem to take near 10x as long to decline to baseline. This statement and interpretation of the LY puff need to be revised. A glutamate puff will likely result in substantial AMPA receptor desensitization.

When we submitted the original manuscript, the only control LY puff recording that I could find used a 20 ms puff, rather than the 2 second puff used for experiments. We have recorded new controls using a comparable 2 second puff and have included one of those experiments in the revised manuscript. These experiments show that the puff solution washes out of the imaged area of the recording chamber in about 15 seconds, similar to the time course of decay of Cx36-GCaMP responses to glutamate puffs. I have modified the results text describing this control (lines 363-367) somewhat, emphasizing that we don’t know whether ion channel or transporter kinetics may shape the gap junction calcium signal.

6) Lines 339-342: it is not clear why synaptic vs non-synaptic NMDA receptors would be expected to have dependence on VGCCs. The logic of this statement is not clear.

This statement was meant to refer to the activation of VGCCs by depolarization induced by activation of synaptic NMDA receptors. In this case, synaptic NMDA receptors at some distance from the gap junction may still elicit a calcium signal that we could detect. In contrast, the non-synaptic NMDA receptors referred to are very close to Cx36 gap junctions and may elicit a calcium signal directly. The statement was not very clear. To clarify the results, I have added a statement that the non-synaptic NMDA receptors on AII amacrine cells are very close to Cx36 gap junctions (lines 405-406) and a clarification to the statement in question (lines 431-434) indicating the calcium detected at Cx36 could come directly through the non-synaptic NMDA receptor channels.

7) The interpretation of Cd2+ effects as evidence for VGCC contributions needs further clarification. In 3H, it’s not clear how the Cd effect points to a contribution of VGCCs when the Cd might just be inhibiting NMDARs. The conclusion might be strengthened with Ca-channel specific blockers. For instance, nifedipine is listed in the methods section, but it’s not apparent that data with nifedipine is included in the manuscript. L-type channels seem likely candidate (and would be mostly blocked by nifedipine), but it’s not clear why data with nifedipine were not included.

We had originally left out the nifedipine data because we did not have enough slices properly paired with control glutamate puff and glutamate + nifedipine to perform a statistical analysis. I have added back some AMPA and AMPA + nifedipine puff data in the population measurements (figure 3 K and L), but again not directly paired. No meaningful statistical analysis can be done on these data due to the slice to slice variability in control responses, but responses on average were smaller than AMPA alone, suggesting a contribution of L-type calcium channels to Cx36-GCaMP responses.

With regard to the NMDA puff experiments using CdCl2, we had hoped that the persistence of puff responses to NMDA in the presence of CdCl2, and the smaller median difference of the inhibition (compared to glutamate puff vs. glutamate + CdCl2) would make the case that NMDA receptors are not fully inhibited by CdCl2, although we understand that some of the effect could be attributed to their partial inhibition.
